# Learning curve and complications of unilateral biportal endoscopy–unilateral laminectomy bilateral decompression for lumbar spinal stenosis

**DOI:** 10.20452/wiitm.2024.17905

**Published:** 2024-11-05

**Authors:** Jiashen Shao, Zihan Fan, Hai Meng, Qi Fei

**Affiliations:** Department of Orthopedics, Beijing Friendship Hospital, Capital Medical University, Beijing, China

**Keywords:** complication, learning curve, lumbar spinal stenosis, unilateral biportal endoscopy, unilateral laminectomy bilateral decompression

## Abstract

**INTRODUCTION:**

The unilateral biportal endoscopic (UBE) technique has been widely adopted for treat‑ ment of lumbar disc herniation and lumbar spinal stenosis. Understanding its learning curve, as well as the factors that influence perioperative complications, is crucial for mastering and effectively learning this technique.

**AIM:**

Our aim was to analyze the learning curve of UBE‑unilateral laminectomy bilateral decompression (ULBD) and risk factors associated with perioperative complications.

**MATERIALS AND METHODS:**

Consecutive patients who underwent UBE from June 2021 to December 2023 at the Department of Orthopedics, Beijing Friendship Hospital, were retrospectively analyzed. Baseline information, perioperative data, and preoperative and postoperative subjective scores were recorded for all patients. The learning curve and identified risk factors for complications were analyzed.

**RESULTS:**

A total of 122 consecutive patients who underwent single‑segment UBE‑ULBD were included in this study. The surgical time curve fitting indicated that the surgeon nearly mastered the technique by the 38th case. Consequently, the cohort was divided into 2 distinct phases: a learning phase (cases 1–38) and a mastery phase (cases 39–122). Operative time, estimated blood loss, and drainage volume were higher in the learning phase group than in the mastery phase group, although hidden blood loss in the learning phase group was lower than in the mastery phase group. The visual analogue scale and Oswestry Disability Index scores at the last follow‑up showed significant improvement in both groups as compared with the preoperative period (P <0.05). Complication rate was 7.9% in the learning phase and 3.6% in the mastery phase. Univariate analysis showed that age, body mass index, alcohol consumption, and estimated blood loss were significantly associated with complication rate.

**CONCLUSIONS:**

UBE is an effective minimally‑invasive spinal endoscopic technique for treating lumbar spinal stenosis, offering short time to achieving surgical mastery and a low complication rate.

## INTRODUCTION 

Spinal stenosis is one of the most common degenerative spinal disorders.^1^^;^^2^^;^^3^ Characteristic lesions include hypertrophy of the ligamentum flavum, hypertrophy of the articular synovial joints, and herniated intervertebral disc.^2^ Decreased spinal canal volume and narrowing of the foramina or lateral recesses caused by the abovementioned lesions can compress nerve roots or the Dural sac, leading to symptoms such as lower back pain, lower limb pain, and intermittent claudication.^2^^;^^4^ Several surgical techniques have emerged that have been widely used, including transforaminal lumbar interbody fusion (TLIF), posterior lumbar interbody fusion, as well as microscopic and endoscopic unilateral laminectomy with bilateral decompression (ULBD).^5^^;^^6^^;^^7^^;^^8^ However, the optimal treatment remains not clearly established, and difficulty in mastering various surgical techniques varies.

In recent years, unilateral biportal endoscopy (UBE) techniques have been widely used in treatment of lumbar disc herniation and lumbar spinal stenosis.^9^;^10^ Several studies have reported relatively satisfactory clinical outcomes for UBE, as compared with other techniques.^11^^;^^12^^;^^13^^;^^14^ In comparison with open surgery, endoscopic ULBD is less stripping and invasive to the paravertebral muscles, and provides a clearer and wider field of view with the assistance of an aqueous medium.^15^In addition, the UBE technique allows a surgeon to have a wider field of view and maneuvering space than uniportal endoscopy.

As an emerging technique, the details of its manipulation and difficulty of mastery have received widespread attention. Spine surgeons are motivated to master the endoscopy technique, but there are still few studies related to the learning curve of the UBE or UBE‑ULBD.^16^^;^^17^ As endoscopic spine surgery has been on the rise in recent years, we intended to provide a reference for the implementation of such surgical modalities by analyzing the learning curve of a single spine surgeon at a spine center. While the experience of a single center or a surgeon may not be fully representative, the results of this study may be valuable to surgeons seeking to incorporate UBE or UBE‑ULBD into their regular surgical practice while ensuring patient safety.

## AIM 

The aim of this study was to analyze the surgical data of patients undergoing UBE‑ULBD at our hospital, and to analyze both the learning curve and the risk factors associated with perioperative complications.

## MATERIALS AND METHODS 

### Patient selection 

Consecutive patients who underwent UBE from June 2021 to December 2023 at the Department of Orthopedics, Beijing Friendship Hospital, were retrospectively analyzed. The surgical approach included unilateral laminectomy with bilateral decompression (UBE‑ULBD). All procedures were performed by the same surgeon, who was experienced in open‑spine surgery but had never performed UBE. The surgeon was a senior orthopedic (spine surgery subspecialty) surgeon who had independently performed more than 800 open lumbar decompression surgeries and completed 6 months of UBE‑related training at several spine centers prior to performing these operations. The first assistants were 1 of 2 qualified spine surgeons.

The inclusion criteria were as follows: 1) patients presenting with low back pain with or without intermittent claudication; 2) magnetic resonance imaging showing stenosis of the central spinal canal, lateral recess, or intervertebral foramen; 3) failure to relieve symptoms after more than 3 months of standardized and systematic conservative treatment; and 4) surgery performed by the same surgeon. We used the following exclusion criteria: 1) simple lumbar disc resection by UBE; 2) operations on more than 2 segments; 3) severe instability or scoliosis of the lumbar spine; 4) lumbar decompression surgery or lumbar interbody fusion surgery at the same level; 5) spinal infection, tumor, or tuberculosis.

### Surgical technique 

**Figure 1:**
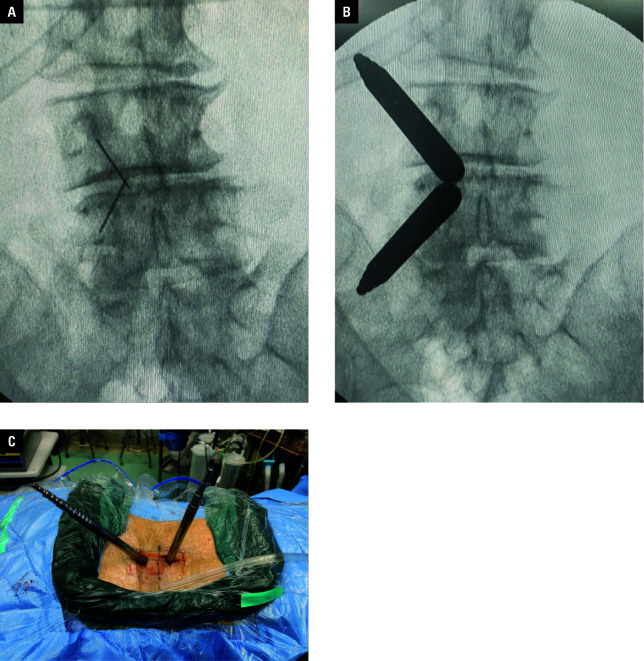
Incision design and instrument placement; **A, B** – subcutaneous localization and channel establishment; **C** – schematic course of unilateral biportal endoscopic surgery

The patients were placed prone on a spine bed under general anesthesia. Under C‑arm fluoroscopy, the surface projections of the midline, intervertebral space level, and pedicles were determined, and the surgical table was adjusted, making the target intervertebral space as perpendicular to the floor as possible. Two 2‑cm incisions were made 1.5 cm superior and inferior to the intervertebral space level in the me‑ dial line of the ipsilateral pedicle Figure 1. The left incision was used as an observation channel and the right incision was used as a working channel. The deep fascia was incised perpendicular to the skin incision. The bilateral channels were dilated using a graduated dilatation cannula, with both dilatation cannulas accessible to and meeting at the vertebral plate and intervertebral space. The surgeon held the spinal endoscope in the left hand and the instrumentation in the right hand. Through both channels, the endoscopic lens and instrumentation met in the aqueous medium. The saline height was adjusted to be at approximately 70–100 cm from the incision.

The soft tissues on the surface of the intervertebral space were handled under radiofrequency probes (BONSS, Jiangsu, China), gradually exposing the inferior margin of the superior lamina, the base of the spinous processes, the articular process joints, and the superior margin of the inferior lamina. The bone (including part of the inferior margin of the lamina, the superior margin of the lamina and the articular process) was partially removed by using a grinder, a bone cutter, and a vertebral plate biting forceps. The ipsilateral ligamentum flavum was resected after exposing its beginning, and the decompression range was up to the inner wall of the pedicle. Part of the base of the spinous process was then removed with a grinding drill. The contralateral ligamentum flavum was decompressed using the contralateral pedicle wall as a reference. Finally, complete resection of the bilateral ligamentum flavum was accomplished. After confirming complete decompression, radiofrequency probes were used for hemostasis. The incision was then closed, and a drain was placed.

### Data collection and analysis 

General information collected about all patients included age, sex, height, weight, underlying medical conditions, surgical segmentation, smoking and alcohol consumption, medication use, and American Society of Anesthesiologists classification.^18^ The operative time, estimated blood loss (EBL), hidden blood loss (HBL), volume of drainage, complications, and preoperative and postoperative subjective scores, including the Oswestry Disability Index (ODI) and the visual analogue scale (VAS) for back and leg, were recorded. Surgical time was calculated from the start of anesthesia to the incision closure. The VAS was used to assess the degree of back and leg pain, and the ODI was used to assess limb function. The data were recorded preoperatively, 3 days postoperatively, and at the last follow‑up.

Cumulative sum (CUSUM) is a statistical method of sequential analysis that has been widely used in health care in recent years.^19^ The learning curve was based on operative time, blood loss, and hidden blood loss, and calculated using the CUSUM analysis. The following formula was used: CUSUM = (Xi–U). Using operative time as an example, Xi represents the operative time of each case, U represents the average operative time of all cases, and n represents the consecutive case number organized chronologically from the earliest to the latest operation date. The learning curves were considered complete when the inflection points appeared in the CUSUM plot. Scatter plots of the results of the CUSUM analyses were plotted using Microsoft Excel 2019 (Redmond, Washington, United States), and the function formulas were obtained through curve fitting. The degree of curve fitting was judged by the coefficient R^2^, the closer R^2^ was to 1, the higher the degree of curve fitting. The model with the highest R^2^ was considered the best‑fitting one. The fitted curve was first derived, and the peak value of the fitted curve was determined according to the value of the slope of the curve to classify the learning stage accordingly. Previous similar studies have used the change in surgical time to delineate phases of learning, and by fitting the surgical time curve, the slope of the curve changed from positive to negative at some point in time. Therefore, the cutoff points for achieving UBE proficiency in surgical technique is usually set at that point.

### Statistical analysis 

R studio 4.4.0 software (R Foundation for Statistical Computing, Vienna, Austria) was used for statistical analysis. The t test or the Mann–Whitney test was used for comparison of continuous variables. The χ^2^ test or the Fisher exact test was used to compare categorical variables. Continuous variables are reported as mean with SD or median with interquartile range. Proportions are reported as frequencies and percentages of the total cohort. *P* values below 0.05 were considered significant.

### Ethics statement 

The experimental protocol was reviewed and approved by the Ethics Committee of Beijing Friendship Hospital (2022KY087). Our study was conducted in accordance with the experimental protocol and the Declaration of Helsinki, and informed consent was obtained from all participants. Apart from routine treatment during hospitalization, there were no other treatments relevant to this study and no additional risks.

**Table 1:** Summary of demographic characteristics

Characteristic		Learning phase (n = 38)	Mastery phase (n = 84)	*P *value
Age, y, mean (SD)		68.4 (11.9)	65.8 (9)	0.17
Sex	Men	12 (31.6)	33 (39.3)	0.41
	Women	26 (68.4)	51 (60.7)	
BMI, kg/m2, mean (SD)		25.3 (4.2)	26.4 (3.4)	0.14
Hypertension	No	13 (34.2)	31 (36.9)	0.77
	Yes	25 (65.8)	53 (63.1)	
Diabetes	No	28 (73.7)	67 (79.8)	0.45
	Yes	10 (26.3)	17 (20.2)	
Coronary artery disease	No	32 (84.2)	75 (89.3)	0.43
	Yes	6 (15.8)	9 (10.7)	
Smoking	No	34 (89.5)	69 (82.1)	0.3
	Yes	4 (10.5)	15 (17.9)	
Alcohol consumption	No	37 (97.4)	75 (89.3)	0.16
	Yes	1 (2.6)	9 (10.7)	
ASA classifications	I–II	28 (73.7)	61 (72.6)	0.99
	≥III	10 (26.3)	23 (27.4)	
Operated segment	L2–L3	1 (2.6)	2 (2.4)	0.9
	L3–L4	5 (13.2)	9 (10.7)	
	L4–L5	27 (71.1)	63 (75)	
	L5–S1	5 (13.2)	10 (11.9)	

Data are presented as number and percentage unless indicated otherwise. Abbreviations: ASA, American Society of Anesthesiologists; BMI, body mass index

## RESULTS 

A total of 122 consecutive patients who underwent single‑segment UBE‑ULBD were included in this study. Mean (SD) follow‑up was 11.6 (2.9) months (range, 8–20 months). There were 45 men and 77 women, at a mean (SD) age of 66.6 (1) years (range, 21–92 years), and a mean (SD) body mass index of 26.1 (3.8) kg/m^2^. The operated segments included L2/L3 (n = 3), L3/L4 (n = 14), L4/L5 (n = 90), and L5/S1 (n = 15) Table 1.

**Figure 2:**
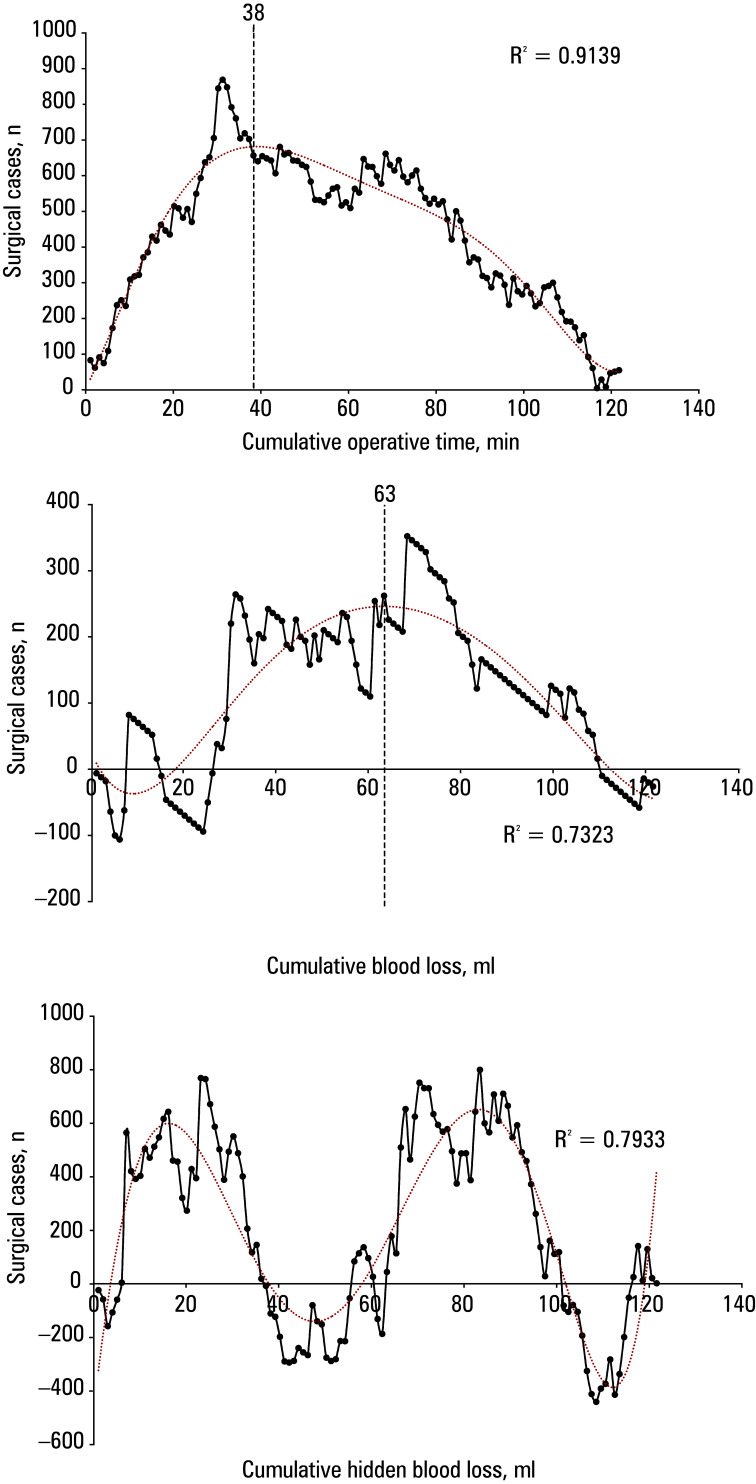
Cumulative sum graph of the operative time (**A**), blood loss (**B**), and hidden blood loss (**C**)

The CUSUM values for surgical time, EBL, and HBL were used to create scatter plots and to fit the curves Figure 2. The results of the curve fit‑ ted for the operative time showed that the slope of the curve changed from positive to negative when the number of surgical cases reached 38 cases. For the comparison of data, we divided the learning curve of 122 UBE cases into 2 phases: the learning phase (case 1–38) and the mastery phase (cases 39–122). Comparison of patient characteristics and perioperative variables between the phases is shown in Table 2. The operative time was longer in the learning phase group than in the mastery phase group (153.3 min vs 129.2 min; *P *< 0.05). The learning phase group had more blood loss than the mastery phase group, but less hidden blood loss than the mastery phase group. There was more drainage in the learning phase group than in the mastery phase group (*P *<0.05). The VAS and ODI scores at the last follow‑up improved in both groups, as compared with the preoperative period (*P *<0.05) Table 2. The complication rates in the learning and mastery phases were 7.9% and 3.6%, respectively. In addition, we plotted fitted curves based on the CUSUM values for blood loss and HBL. In terms of the change in blood loss, when the cumulative number of surgical cases of the operator reached 63, the blood loss was significantly limited. In terms of reduction in HBL, 2 significant thresholds occurred, when the number of surgical cases reached 16 and 83, respectively.

A total of 6 postoperative complications occurred in all patients (4.9%). Therefore, we di‑ vided the cohort into the complication and noncomplication groups. We analyzed the factors associated with the complications in univariate and multivariate analyses. The results suggested that age, BMI, alcohol consumption, and EBL were associated with the occurrence of complications Table 3.

**Figure 3:**
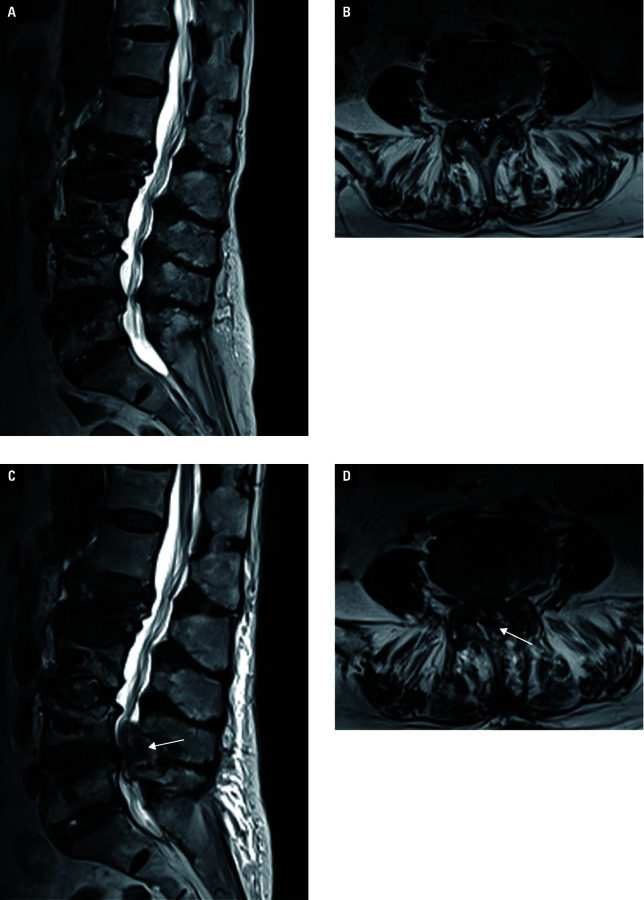
A case of epidural hematoma after unilateral biportal endoscopy (UBE)–unilateral laminectomy bilateral decompression. An elderly woman underwent UBE for lumbar spinal stenosis (**A, B**). On the second postoperative day, she suddenly developed radiating pain in the right lower extremity with a visual analog score of 9. Magnetic resonance imaging showed formation of an epidural hematoma (**C, D**; white arrow). The patient did not undergo further hematoma debridement owing to her poor physical condition. After 2 months of conservative treatment, the symptoms were significantly relieved.

Details of all patients with complications are shown in Table 4 . A total of 3 patients had intraoperative dural tears due to severe adhesions, all of which were repaired intraoperatively with absorbable dural patches, and no massive cerebrospinal fluid leaks, pseudomeningeal cysts, or meningitis were detected postoperatively. One patient from each of the learning (26th) and mastery phases (86th) presented with residual postoperative symptoms resulting from inadequate decompression. The patient 26 experienced a moderate improvement of symptoms after taking conservative treatment, while the other one (86th) improved after posterior lumbar interbody fusion surgery 3 months postoperatively. A patient with epidural hematoma (34th) presented with worsening radiating pain in the right lower extremity on the second postoperative day, and the symptoms relieved within 2 months after conservative treatment Figure 3.

## DISCUSSION 

Surgery is an  effective treatment modality for lumbar spinal stenosis whose symptoms persist after conservative treatment. The primary purpose of surgery is to relieve nerve compression and enlarge the capacity of the spinal canal. There are several surgical procedures used to treat lumbar spinal stenosis, including open surgery, microsurgery, and percutaneous endoscopic surgery. The use of the UBE technique in spinal disorders has become more widespread in recent years. According to the results of a recent meta‑analysis involving 528 patients who underwent UBE, both VAS and ODI scores demonstrated significant improvement following UBE surgery when compared with preoperative values. These findings underscore the favorable clinical outcomes associated with UBE.^20^ The main features of this technique include a direct creation of a channel through the skin, thus providing a wide range of instrumentation movement, complete decompression, and less bone resection. Some studies have reported that the ipsilateral and contralateral facet joint preservation rate of UBE‑ULBD is 78% and 85%, respectively.^21^ Therefore, the UBE technique also has a smaller impact on lumbar spine stability. As an emerging technique, UBE requires a certain period to master it. The learning curve reflects the rate at which this technique is mastered over a given period of time, and is indicative of the number of surgical cases required to achieve proficiency. Although the learning curve is affected by certain subjective variables, it serves to summarize the experience and provide a reference.

The CUSUM analysis is a statistical metric for quantitatively evaluating learning curves, and it can provide physicians with timely graphical feedback.^22^ The CUSUM graph accurately represents the temporal relationship between the number of surgical cases in chronological order and the surgeon’s ability to perform a specific surgical task. This graphical representation is a powerful formative assessment for both physicians and proficiency throughout the training process. In this study, we used the CUSUM analysis to determine the learning curve in terms of operative time, blood loss, and HBL. Although there is some variation between the 3 charts, we can conclude that for a surgeon experienced in open and minimally‑invasive spine surgery, up to 40 surgical cases may be sufficient to master the technique.

Many studies have reported the learning curve for other spinal surgeries. Nandyala et al^23^ studied the learning curve for the minimally‑invasive TLIF procedure and found that after 33 cases, the operative time gradually stabilized, proving that the surgical technique had been mastered. Xu et al^17^conducted a preliminary study of the  learning curve of UBE surgery, and found that the operative time gradually stabilized and began to decrease from the 54th case. Choi et al^24^ reported that the operative time remained stable after the 36th UBE procedure. Kim et al^25^ considered that at least 34 procedures

**Table 2:** Surgical variables and clinical outcome

Characteristic	Learning phase (n = 38)	Mastery phase (n = 84)	*P *value
Surgical time, min	153.3 (44)	129.2 (34.5)	0.003
Estimated blood loss, ml	62.4 (42.3)	53.1 (31.5)	0.18
Hidden blood loss, ml	182.4 (121.8–256.1)	197.8 (123.7–286.9)	0.86
Drainage volume, ml	18 (9–37)	50 (35–90)	0.001
Total complication rate, n (%)	3 (7.9)	3 (3.6)	0.66
Preoperative ODI	45.1 (2.1)	45.4 (1.3)	0.44
Early ODI	25.3 (1.3)	24.9 (2.2)	0.25
Late ODI	22.3 (1.4)	22 (2.1)	0.43
Preoperative VAS back	6.6 (1)	7.2 (1.1)	0.007
Early VAS back	3.1 (0.6)	3.3 (3.2)	0.12
Late VAS back	2.2 (1.2)	2.7 (3.5)	0.07
Preoperative VAS leg	6.3 (1)	7.2 (1.1)	0.001
Early VAS leg	3.1 (0.8)	3.4 (0.8)	0.03
Late VAS leg	3.2 (0.7)	3.6 (0.9)	0.02

Data are presented as mean (SD) or median (interquartile range) unless indicated otherwise. Abbreviations: ODI, Oswestry Disability Index; VAS, Visual Analogue Scale

were needed to sufficiently master UBE lumbar interbody fusion. The CUSUM analysis of the operative time in our study showed that the cutoff point for mastery of the UBE‑ULBD technique was 38 cases. The mean (SD) operative time in the mastery phase was approximately half an hour shorter than in the learning phase (153.3 [44] min vs 129.2 [34.5] min). Also, the mean blood loss and drainage volume were lower in the mastery phase than in the learning phase. Usually, operative time is a critical indicator for evaluating a surgeon’s ability to master this technique, but theoretically the learning curve should be evaluated in terms of the procedure safety and health benefits to the patient, rather than solely in terms of the level of surgical proficiency. Therefore, when determining the learning curve, it is important to consider not only operative time but also the occurrence of complications. In our study, there was no significant difference in the complication rate between the learning and mastery phases (7.9% vs 3.6%).

We identified age, BMI, alcohol consumption, and EBL as factors impacting the onset of complications. Several studies have yielded similar conclusions. For example, Kim et al^26^ found that female sex, advanced age (over 70 years), preoperative anticoagulant medication use, and intraoperative hydration pump use were independent risk factors for postoperative epidural hematomas. We suggested that advanced age and obesity may have some implications for surgical maneuvers. For example, elderly patients often have a history of severe adhesions between the ligamentum flavum and the epidural and more calcification of the intervertebral discs, which can severely affect intraoperative maneuvers. Obesity can also impede the endoscopic maneuvers due to a thick layer of subcutaneous fat. All these factors may lead to intraoperative adverse events and postoperative complications. We did not perform a multivariable regression analysis of risk factors for individual complications, due to a limited number of the complications, but such an analysis should be performed in future studies.

Several previous studies have discussed the characteristics of the learning curve for full spinal endoscopy and the cutoff point required to achieve technical proficiency. A systematic review including 6 studies that applied interlaminar approaches for spinal endoscopic procedures indicated that the mean (SD) value of the cutoff point for the learning curve was 22.17 (12.4) cases (range, 10–43 cases).^27^ These learning curves were determined primarily based on the operative time, which was shorter in the patients operated later than those operated earlier (*P *<0.05). Also, no significant differences were found in pain scale, functional outcome, operative failure, or complication rates in the patients before and after the cutoff point. A comparative study of learning curves for percutaneous endoscopic transforaminal discectomy performed by surgeons with different levels of professional experience found that the reoperation rate within 1 year was higher in the group of junior surgeons than senior ones.^28^ The results of these studies suggest that the level of mastery is not only manifested by significantly shorter operative time, but more so in terms of operative efficacy and reoperation rate. In our study, no patient showed short‑term recurrence during follow‑up. Longer follow‑up for these patients is essential.

There are some limitations to our study. First, the surgeon performed the UBE‑ULBD procedure after initial attempts of the UBE technique in some simple cases, and therefore, the cutoff

**Table 3:** Univariate analysis of postoperative complications

Characteristic		Without complication (n = 116)	Complications (n = 6)	*P *value
Sex	Men	44 (37.9)	1 (16.7)	0.31
	Women	72 (62.1)	5 (83.3)	
Age, y, mean (SD)		66.3 (0)	77.2 (4.8)	0.007
BMI, kg/m2, mean (SD)		26.1 (3.8)	29.7 (2.8)	0.03
Hypertension	No	42 (36.2)	1 (16.7)	0.35
	Yes	74 (63.8)	5 (83.3)	
Diabetes	No	91 (78.4)	3 (50)	0.13
	Yes	25 (21.6)	3 (50)	
Coronary artery disease	No	102 (87.9)	4 (66.7)	0.16
	Yes	14 (12.1)	2 (33.3)	
Anemia	No	107 (92.2)	6 (100)	0.99
	Yes	9 (7.8)	0 (0)	
Smoking	No	97 (83.6)	4 (66.7)	0.3
	Yes	19 (16.4)	2 (33.3)	
Alcohol consumption	No	106 (91.4)	3 (50)	0.007
	Yes	10 (8.6)	3 (50)	
History of anticoagulation medication	No	112 (96.6)	5 (83.3)	0.15
	Yes	4 (3.4)	1 (16.7)	
ASA classifications	I–II	84 (72.4)	3 (50)	0.47
	≥III	32 (27.6)	3 (50)	
Operated segment	L2–L3	3 (2.6)	0	0.48
	L3–L4	13 (11.2)	1 (16.7)	
	L4–L5	85 (73.3)	5 (83.3)	
	L5–S1	15 (12.9)	0	
Tranexamic acid	No	56 (48.3)	3 (50)	0.93
	Yes	60 (51.7)	3 (50)	
Surgical time, min, mean (SD)		136.5 (39)	170 (45.9)	0.056
Estimated blood loss, ml, mean (SD)		56 (35.9)	100 (31.6)	0.01

Data are presented as number and percentage unless indicated otherwise.

Abbreviations: see Table 1

**Table 4:** Details of complications

Complication type	Number of complications	Procedure number
Dural tear	3	18, 78, 102
Symptomatic residue	2	26, 86
Postoperative hematoma	1	34

point of the learning curve may have been affected. This problem exists in many similar studies, for example, in the early stage of the application of a certain technique, the surgeons tend to select cases with typical unilateral symptoms, clear indications for surgery, and a relatively minor degree of complexity, and then gradually conduct more challenging operations after the technique has been mastered. Second, due to heterogeneity of clinical cases, different obstacles are encountered when performing surgery. For example, cases with severe adhesions may be more challenging and may need a prolonged operative time. These problems are; however, typical of all such studies. Third, only the patients operated on by a single surgeon were included in this study, and no comparison was made between the learning curves of surgeons at different levels. When performing UBE procedures, the seniority of the surgeon and their endoscopic experience affect the learning cycle. Fourth, the small number of patients who experienced complications may affect the statistical power of the analysis of risk factors related to the complications. However, this appears to be a common limitation in many similar studies. The primary reason is that the UBE technique is still in its early stages of adoption in China, and is performed less frequently in most medical institutions. Additionally, minimally‑invasive lumbar spine surgery techniques, such as UBE, are associated with a relatively low complication rate, as compared with traditional open surgery. Despite these limitations, we believe the results of this study can serve as a valuable reference for early implementation of this technique.

## CONCLUSIONS 

As an effective minimally‑invasive spinal endoscopic technique for the treatment of lumbar spinal stenosis, the UBE procedure requires approximately 38 cases to overcome the learning curve. When the learning curve is successfully overcome, the technique offers the advantages of minimally‑invasive nature, flexible and efficient maneuvers, and rapid postoperative recovery. We evaluated the learning curve of the same surgeon using the UBE‑ULBD technique in lumbar spinal stenosis using the CUSUM cumulative analysis. The results showed that after 38 cases, the surgeon could achieve a more proficient and stable manipulation level, and could significantly shorten the operation time and improve patient satisfaction. Moreover, we found that characteristics such as obesity, advanced age, and alcohol abuse were associated with the onset of complications, and these are suggestive for preoperative screening of high‑risk patient populations.
